# Twitter as a Potential Disaster Risk Reduction Tool. Part IV: Competency-based Education and Training Guidelines to Promote Community Resiliency

**DOI:** 10.1371/currents.dis.ce3fad537bd666770a649a076ee71ba4

**Published:** 2015-06-29

**Authors:** Violet Yeager, Guy Paul Cooper, Frederick M. Burkle, Italo Subbarao

**Affiliations:** College of Osteopathic Medicine, William Carey University, Hattiesburg, Mississippi, USA; College of Osteopathic Medicine, William Carey University, Hattiesburg, Mississippi, USA; Harvard Humanitarian Initiative, Harvard University, Cambridge, Massachusetts; The Woodrow Wilson International Center for Scholars, Washington, DC, USA; College of Osteopathic Medicine, William Carey University, Hattiesburg, Mississippi, USA

**Keywords:** Communications, Competencies, Disaster risk reduction, Health Informatics, Prevention and preparedness, Public Health, Resiliency, Social media, Twitter

## Abstract

Twitter can be an effective tool for disaster risk reduction but gaps in education and training exist in current public health and disaster management educational competency standards.  Eleven core public health and disaster management competencies are proposed that incorporate Twitter as a tool for effective disaster risk reduction.  Greater funding is required to promote the education and training of this tool for those in professional schools and in the current public health and disaster management workforce.

## Introduction

The previous three parts in this series demonstrate that Twitter has the potential to be an effective and efficient community-based communications tool for disaster risk reduction and management.^1^^–^ 3 The skills required for people to utilize Twitter effectively during a disaster or public health emergency range from effective tweet-based communication, including modifiers and hashtags, computer programming, health informatics, and statistical analysis.^1^^–^ 3 Some applications of this tool lie within the nascent domain of public health informatics. This tool, if properly harnessed, can aid public health providers in disaster risk reduction and management. Within the context of dynamic innovations in global communication and technology, public health education must evolve and adapt to meet the needs of the population that public health officials serve. This must be accomplished while effectively and efficiently staying true to the competencies and standards defined by the public health community as displayed with the “10 Essential Public Health Services.”^4^ Twitter and its informatics base has the potential to be integrated across disciplines and within all levels of public health infrastructure and social protection programs

## Concepts

**COMMUNITY ENGAGEMENT FOR DISASTER PLANNING AND MITIGATION**

Twenty-first century public health workers and emergency managers need to be able to communicate with their communities in order to achieve resiliency. Social media now plays an ever greater role in communicating with populations in all phases of the disaster cycle: prevention, preparedness, response, and recovery. These studies demonstrate that for all disaster planning phases, public health workers and emergency management teams should initially focus on identifying and recruiting social media savvy individuals that have large followers in the local region or among the targeted and potentially at-risk population in building ‘disaster response follower’ bases. Using data from the 2013 Hattiesburg F4 tornado, user followers along with the Klout score were shown to be the strongest pre-storm significant indicators associated with exponential message distribution, allowing for the social media community leaders or superspreaders to be identified while allowing them to remain anonymous.^1^^–^ 3

This ability to identify community leaders enables public health officials and disaster managers to facilitate partnerships through recruitment and mobilization efforts within the community. The average Twitter follower has 208 followers, but local superspreaders, as demonstrated in Parts I-III, show the top 100 rated users have an average of 1,000 Twitter followers which is 5 times the average follower.^1^^–^ 3 If superspreaders (or community leaders) are provided proper competency-based training, this process has the potential for rapid and effective dissemination of information to a targeted population group via disaster and public health officials. Additionally, increasing the followers for superspreaders, community leaders, disaster response teams, and public health officials that use Twitter would allow for enhanced education, information dissemination, and empowerment of population groups previously inaccessible prior to the influence of social media. Past studies found that social media is changing current communication patterns.^5^ Community leaders and disaster managers, by engaging with these new social media communication tools have the potential to improve health outcomes by optimizing communication within each disaster phase.^6^ To date, a working relationship with social media and the tools it offers has not been a priority in the emergency management profession.

Burkle and Greenough emphasize the need for continued community engagement throughout the disaster cycle and the inherent challenges of monitoring public health outcomes in a crisis environment.^7^ The impact of compromised public health infrastructure and systems on health consequences defines and greatly influences the manner in which disaster are observed, planned for, and managed, especially those that are geographically widespread, population dense, and prolonged. What may first result in direct injuries and deaths may rapidly change to excess indirect morbidity and mortality as essential public health resources are destroyed, deteriorate, or are systematically denied to vulnerable populations.”^7^ Community engagement with Twitter can greatly enhance the immediate situational awareness leading to a more directed response and recovery actions. The “prepared community concept” popular in Australia has led to more “effective and efficient” response and recovery phases of the disaster cycle.^8^

**COMMUNITY ENGAGEMENT FOR DISASTER RESPONSE AND RECOVERY**

Disaster notification resources must adapt and evolve to the reality that the at-risk populations may not have access to local television, radio, or be within range of warning sirens during a disaster. This confirms the need for practical applications of social media assets. Currently, much of the social media research in the realm of public health is placed on the academic perspective of investigation where the emphasis is focused on proving an application’s worth in controlled environments or groups. This limits the practical application, even for trained public health and disaster managers, as the application must be broadened to allow for the targeting of a population group while accounting for behaviors unique to that population. In this series, Twitter has shown unique features with the potential to be utilized for practical application by disaster officials and public health managers across and within the disaster cycle.^1^^–^ 3

Parts I-III highlight the features of Twitter that allow for it to be effectively utilized by disaster and public health managers as a publicly accessible and cost-effective network for instantaneous dissemination. Additional features include the ability to collect accurate and reliable data both retrospectively and in real-time, and the capacity to target a population group, all while allowing those who use it to maintain anonymity. These qualities allow for an easy and practical application in all phases of disaster management but call for additional education, training and program development before any application of this technology in monitoring and health surveillance can be effectively operationalized.

**COMPETENCY BASED EDUCATION AND TRAINING**

Current education and training programs for the public health workforce for the 21st century do not necessarily prioritize the competencies and skills that are required to best utilize technology and social media interventions including Twitter. The Core Competencies for Public Health Professionals (Core Competencies) are defined by skill sets for broad practice of public health defined by “10 Essential Public Health Services”.^4^^,^ 9 These provide a framework for National Public Health Performance Standards (NPHPS) and public health educators.^10^ The competencies for public health professionals are developed by the Council on Linkages Between Academia and Public Health Practice (Council on Linkages), which is funded by the Centers for Disease Control and Prevention (CDC) and is supported by the Public Health Foundation consisting of a collaboration of 20 national organizations. They are designed to improve public health education, training, research, and workforce development through evidence-based practice of public health.^11^ These competencies are dynamic and constantly evolving to meet the needs of the public health field and the population groups the health providers serve.^12^ Yet, the current competencies are limited in providing direction and standards on communication within technology and social media, such as Twitter. If competencies are provided to the public health community, the proper infrastructure, training, and policy can be developed to harness the potential of this global communication tool through technology while a public health alliance works to instill standards to ensure proper ethical, legal and transparent applications as the technology evolves in the future.

Past studies proposed that disaster-specific public health preparedness competencies should place greater value on the use of social media skills for relevant risk communication.^13^^,^ 14 However, these studies do not fully appreciate the requirements for competency for global adaptation of this tool within an ever-changing multidimensional technological environment across the world. For operational application of social media, performance competency is vital to permit ethical and sustainable implementation of technology. It would allow for it to be taught at all levels and fields of public health. Standards would progress the field of public health in the age of technology while aiding in efficiently carrying out the basic public health functions of assessment, policy development, and assurance. Additionally, this would allow for cross-disciplinary and multidisciplinary adoption of standards.

**Domain:** Public Health and Disaster Informatics Competency Recommendations Include:

1) Ethical and effective communications using Twitter and other social media outlets during all phases of the disaster life cycle.

2) Effectively modify tweets and social media messages to positively enhance dissemination of information with cross-cultural competency to meet the needs of a target population group, allowing for better information, education, and empowerment of the population group across the disaster life cycle.

3) Effectively program data sets to filter and triangulate data for a targeted population during all phases of the disaster life cycle.

4) Facilitate the use of technology and social media, such as Twitter, to aid in community mobilization and partnerships to identify and solve health problems during all phases of the disaster life cycle.

5) Apply appropriate statistical analyses and data retrieval techniques to allow for accurate and reliable data recovery during all phases of the disaster life cycle.

6) Ethically monitor, investigate, and survey health problems and hazards with real-time surveillance systems and databases leveraging Twitter and other social media outlets during all phases of the disaster life cycle.

7) Ensure competency-trained access of technology and social media outlets such as Twitter for all levels of disaster managers and public health providers during all phases of the disaster life cycle.

8) Emphasize the importance of translating academic research to practical public health application during all phases of the disaster life cycle.

9) Develop and evolve ethical policies, laws, and regulations to protect health and ensure safety of all who use technology and social media such as Twitter during all phases of the disaster life cycle.

10) Evaluate and monitor the effectiveness, accessibility, and quality of population-based health information obtained through technology and social media such as Twitter during all phases of the disaster life cycle.

11) Apply transparency, systemic, consistent, comprehensive, accurate, and reliable data collection methods from social media, such as twitter, for assessment, development, implementation, evaluation, and reassessment/modification of public health programs and initiatives during all phases of the disaster life cycle.

Communication is fundamental to public health.^14^ These competencies must be embedded in public health schools and become part of the foundation for today’s evolving public health education and disaster management. Public health schools should take the lead in training future public health and disaster managers to acquire these skill sets to meet the needs of the population they serve. Program managers are expected to consistently target and meet their population group needs while promoting initiatives allowing for cost efficiency and maintaining integrity of the public health program; and allow for adoption of those needs both with direct interest while sharing technological advances with others in the field.^15^^,^ 16 For these expectations to occur, new skill sets must evolve along with technology.

**EDUCATION AND TRAINING TO PROMOTE RESILIENCY** ****

For implementation and sustainability of this technology in the field of public health and disaster management funding must be provided at all levels. Funding would allow for training and education in public health schools linked to communities to meet these competency requirements. Funding for state and local public health must be provided for real-time monitoring, disease surveillance, program monitoring and evaluation, and for training and education of public health workers and emergency managers responsible for disaster planning in their communities. Additionally, funding would be required for readiness assessment exercises to assess training and monitor standards.^11^^,^ 13 Each would allow for communities to be prepared for potential threats while implementing the most up-to-date efficient tools. Due to the unique criteria required for disaster response the CDC plays a key role in ensuring state and local agencies are prepared for disaster preparedness and are the main source of funding from the Public Health Emergency Preparedness cooperative agreement. Unfortunately, this funding is declining.^17^ In light of this decrease it becomes crucial to prioritize funding for the development of a sustainable infrastructure, especially at the local level, which might leverage the greater efficiency and cost-effectiveness provided by social media.

## Limitations

Competency-based content has its limitations. There are different ways in which one improves their skills: through practice of those skills, through professional development to practice in a competent manner, and to properly identify indicators by which their practice may be recognized as having achieved certain levels of proficiency. Twitter users come to this task in disaster risk reduction with daily experience in its usage. This project speaks only of honing that development in terms of expectations around a specific disaster event. There remains a paucity of experience other than what is described in this study. The competencies listed represent guidance the authors consider today as core requirements. Competencies will change as the technology improves, as Twitter is used in a variety of disaster and crisis events, as the practice environment evolves, and as professional standards and legal regulations of practice are determined. The competencies presented here provide a general framework that should allow for the launching of the structured education and training to progress.

## Conclusions

Twitter, if properly harnessed, can aid public health providers in providing low cost and effective disaster risk reduction and management. This process has the potential of meeting all the basic public health functions of assessment, policy development, and assurance. It is also likely that the above competencies may be broadened for daily public health functions and tasks. Planners must not lose sight that this process should be accomplished as part of community planning especially in disaster-prone communities and regions worldwide. The evidence provided by these studies originated from a disaster prone area in Mississippi, yet demonstrates the first steps of what can be accomplished on a wider scale.

## Competing Interests

The authors have declared that no competing interests exist.
